# Study on suitable analysis method for HIV-1 non-catalytic integrase inhibitor

**DOI:** 10.1186/s12985-020-01476-x

**Published:** 2021-01-13

**Authors:** Ki Hoon Park, Minjee Kim, Seoung Eun Bae, Hee Jung Lee, Kyung-Chang Kim, Byeong Sun Choi, Young Bong Kim

**Affiliations:** 1 Department of Bio-Industrial Technologies, College of Animal Bioscience and Technology, Konkuk University, Seoul, Korea; 2 Department of Biomedical Science and Engineering, College of Animal Bioscience and Technology, Konkuk University, Seoul, Korea; 3 Division of AIDS, Center for Immunology and Pathology, Korea National Institute of Health, Osong, Chungcheongbuk Korea

**Keywords:** Integrase, Anti-HIV drug, HIV-1, Non-catalytic integrase inhibitor, Screening method

## Abstract

**Background:**

Integrase (IN) is an essential protein for HIV replication that catalyzes insertion of the reverse-transcribed viral genome into the host chromosome during the early steps of viral infection. Highly active anti-retroviral therapy is a HIV/AIDS treatment method that combines three or more antiviral drugs often formulated from compounds that inhibit the activities of viral reverse transcriptase and protease enzymes. Early IN inhibitors (INIs) mainly serve as integrase strand transfer inhibitors (INSTI) that disrupt strand transfer by binding the catalytic core domain of IN. However, mutations of IN can confer resistance to INSTI. Therefore, non-catalytic integrase inhibitors (NCINI) have been developed as next-generation INIs.

**Methods:**

In this study, we evaluated and compared the activity of INSTI and NCINI according to the analysis method. Antiviral activity was compared using p24 ELISA with MT2 cell and TZM-bl luciferase system with TZM-bl cell. Each drug was serially diluted and treated to MT2 and TZM-b1 cells, infected with HIV-1 AD8 strain and incubated for 5 and 2 days, respectively. Additionally, to analyze properties of INSTI and NCINI, transfer inhibition assay and 3′-processing inhibition assay were performed.

**Results:**

During screening of INIs using the p24 ELISA and TZM-bl luciferase systems, we found an inconsistent result with INSTI and NCINI drugs. Following infection of MT2 and TZM-bl cells with T-tropic HIV-1 strain, both INSTI and NCINI treatments induced significant p24 reduction in MT2 cells. However, NCINI showed no antiviral activity in the TZM-bl luciferase system, indicating that this widely used and convenient antiretroviral assay is not suitable for screening of NCINI compounds that target the second round of HIV-1 replication.

**Conclusion:**

Accordingly, we recommend application of other assay procedures, such as p24 ELISA or reverse transcription activity, in lieu of the TZM-bl luciferase system for preliminary NCINI drug screening. Utilization of appropriate analytical methods based on underlying mechanisms is necessary for accurate assessment of drug efficacy.

## Background

Following the first reported cases of acquired immune deficiency syndrome (AIDS) in 1983, AIDS-associated deaths were gradually identified worldwide with increasing prevalence until 2004. However, by 2017, the death rate was reduced by 52% [1]. Due to the expansion of antiretroviral therapy and a consequent decline in new HIV infection cases [2–4].

Antiretroviral drugs are divided into four classes: entry/fusion inhibitor, reverse transcription inhibitor (RTI), integrase inhibitor (INI), and protease inhibitor (PI) [5, 6]. Highly active antiretroviral therapy (HAART), a standard HIV/AIDS treatment method, is a cocktail therapy combining three or more antiretroviral drugs that act on different targets [7]. HAART generally comprises three drugs, specifically, two nucleoside reverse transcription inhibitors (NRTIs) with one non-nucleoside reverse transcriptase inhibitor (NNRTI) or PI (2 NRTIs + NNRTI or 2NRTIs + PI) [8]. Sometimes, RTI + PI combinations are used with INI or entry inhibitor or as alternative treatment options (RTI + PI + INI or RTI + PI + entry inhibitor) [8]. HAART effectively reduces the viral load and facilitates significant recovery of immune functions in HIV/AIDS patients, increasing the survival period by more than 7–10 years or even longer compared to single drug-treated patients [7–9]. However, continued efforts to design more effective novel anti-HIV drugs are urgently required to combat the emergence of highly mutagenic HIV strains and continuous drug resistance [10]. Following the initial approval of zidovudine as a therapeutic NRTI by the US Food and Drug Administration (FDA) in 1987, various anti-HIV drugs have been developed as INI, starting with raltegravir (RAL) in 2007 followed by elvitegravir (EVG) and dolutegravir (DTG) [9, 11, 12].

The integrase (IN) protein plays an important role in transferring viral DNA to the host nucleus in the HIV replication process and serves as an important pharmacological target for next-generation anti-HIV drugs [13, 14]. Integration can be classified into two steps: (1) '3′ processing' that refers to the process of cutting dinucleotides at both ends of viral DNA for formation of a pre-integration complex (PIC) by combining with IN and (2) the strand transfer step whereby PIC is transported into the host nucleus by IN and combined with lens epithelial-derived growth factor/p75 (LEDGF/p75) in chromosomal DNA to integrate viral DNA [14–17]. RAL, EVG, and DTG are strand transfer inhibitors (INSTI) that recognize and bind the catalytic core domain (CCD) of integrase and block this process [12, 13, 17, 18].

In 2014, BI 224436, the first approved non-catalytic site integrase inhibitor (NCINI), was developed by Boehringer Ingelheim (Canada) Ltd. In contrast to INSTIs, BI 224436 binds a highly conserved allosteric site in CCD of IN to induce conformational changes in the catalytic site, thus disrupting interactions of IN with long-terminal repeat (LTR) DNA, and is additionally reported to inhibit 3′-processing [13, 19, 20]. NCINIs, a new class of INI drugs, can overcome the problems of INSTI-resistant viruses based on its different inhibition mechanism. Specifically, INSTIs directly inhibit IN-LEDGF/p75 interactions while NCINIs bind CCD to inhibit IN-LTR DNA generation [13, 20].

We were commissioned by a domestic pharmaceutical company to evaluate the efficacy of a novel NCINI candidate but were unable to verify antiviral activity using the TZM-bl luciferase system. In view of this finding, we attempted to develop a suitable method for accurately assessing the antiviral efficacy of NCINI.

To achieve optimal results, a drug efficacy test should always take into account the biological mechanism. Comparative evaluation of the efficacy of INSTI and NCINI compounds using various in vitro methods in this study facilitated the identification of a novel system appropriate for screening of NCINIs.

## Methods

### Cells and viruses

MT2 cells were maintained in Roswell Park Memorial Institute 1640 medium (RPMI1640, HyClone, Logan, UT) containing 10% fetal bovine serum (FBS, Invitrogen, Carlsbad, CA) and 1% penicillin/streptomycin (P/S, gibco, Waltham, MA). TZM-bl and HeLa cells were maintained in high-glucose Dulbecco’s Modified Eagle's medium (DMEM, HyClone, Logan, UT) containing 10% FBS and 1% P/S. All cell lines were incubated at 37 °C with 5% CO_2_. To produce the infectious HIV-1 AD8 strain, 20 μg pNL4.3(AD8) clone was transfected into HeLa cells using iN-fect™ (iNtRON Biotechnology, Seongnam, Korea). After 48 h of culture, culture media were harvested and centrifuged for 2000 rpm (1344 rcf) at 5 min for removal of cell debris. Harvested viruses were stored at − 80 °C. Infectious virus titers were determined based on 50% tissue culture infectious dose (TCID_50_) according to the endpoint method of Reed and Muench (1938).

### Drugs

RAL, DTG and EVG were kindly provided by the New Drug development team, R&D center, ST Pharm (Seoul, Korea) and BI 224436 by Professor Baek Kim (School of Medicine Health Science Research Building, Emory University, Atlanta, GA).

### Measurement of cell cytotoxicity of anti-HIV drugs

To determine the cytotoxicity of anti-HIV drugs, cell viability was assessed via the water-soluble tetrazolium salt (WST) method using an EZ-Cytox kit (Daeil Lab Service, Seoul, Korea) according to the manufacturer’s instructions. Briefly, TZM-bl and MT2 cells were seeded on 96-well cell culture plates at a density of 1 × 10^4^ cells/well and cultured overnight. Cells were treated with serial dilutions of each drug (two-fold dilutions from 50,000 to 2.54 nM). On days 2 and 5 of incubation, 10 μl EZ-Cytox solution was added to each well and incubated for 2 h, followed by spectrophotometric measurement of absorbance at 540 nm. The CC_50_ value of samples was defined as the concentration inducing 50% cell death.

### Enzyme-linked immunosorbent assay for p24

ELISA was conducted to detect HIV-1 p24 for assessment of antiviral activity using a HIV-1 p24 ELISA kit (XpressBio, Frederick, MD). To this end, MT2 cells seeded on a 96-well cell culture plate at a density of a 1 × 10^4^ cells/well were infected with 500 TCID_50_ of HIV-1 AD8 strain. Each drug was serially diluted threefold (from 10,000 to 0.51 nM) for treatment of cells. After five days of culture at 37 °C with 5% CO_2_, 100 μl of culture medium was harvested and supernatant obtained by centrifugation for 5 min at 5000 rpm (8400 rcf), with storage at − 80 °C. Cell culture media of non-infected and infected cells without INI treatment were set as the negative and positive control, respectively. The ELISA procedure was conducted according to the manufacturer’s instructions. Briefly, samples were mixed with 20 μl lysis buffer, 200 μl aliquots pipetted into a microplate, and incubated for 1 h at 37 °C. After incubation, the contents of the wells were aspirated and microtitration plates washed six times with 350 μl wash buffer. Each well was treated with 100 μl detection antibody for 1 h at 37 °C, which was subsequently removed by washing under the same conditions. An aliquot (100 μl) of streptavidin-HRP conjugate was added into each well, followed by incubation at room temperature for 30 min. After washing under the same conditions, 100 μl substrate solution was immediately dispensed into each well and incubated for 30 min at room temperature with protection from direct sunlight. For termination of the reaction, 100 μl stop solution was added to each well and absorbance values at 450 nm immediately read using a microplate reader.

### Antiviral activity test using the TZM-bl cell system

TZM-bl cells were seeded on 96-well cell culture plates at a density of 1 × 10^4^ cells/well and cultured overnight. Each drug was twofold serially diluted from 10,000 to 0.15 nM and treated to cells. After a 30 min incubation period, viral infection was performed with 500 TCID_50_ of HIV-1 AD8. Cells were cultured for 48 h after infection and luciferase activity measured using Beetle-Lysis Juice (PJK GmbH, Kleinblittersdorf, Germany) according to the manufacturer’s instructions. Briefly, the medium was removed, and cells washed three times with 200 μl PBS. Next, 100 μl Beetle-Lysis Juice containing luciferin and ATP were added to each well and incubated for 5 min with protection from sunlight. Subsequently, luciferase activity was measured using a micro beta counter (PerkinElmer, Waltham, MA) after transferring solutions to a white 96-well plate. Anti-HIV efficacy of drugs was determined based on reduced expression of luciferase relative to the virus-only treatment group.

### Strand transfer inhibition assay

The strand transfer assay was performed using the HIV-1 integrase assay kit (XpressBio, Frederick, MD) according to the manufacturer’s instructions. Briefly, 100 μl of 1X donor substrate DNA (DS DNA) solution was added to each well and incubated for 30 min at 37 °C. The liquid was aspirated from the wells and washed 5 times with 300 μl wash buffer, followed by incubation with 200 μl blocking buffer per well for 30 min at 37 °C. Following aspiration of the liquid, wells were washed three times with 200 μl reaction buffer. Next, 100 μl IN enzyme solution was added to each well and incubated under similar conditions followed by removal of liquid and three washes with 200 μl reaction buffer. Each test sample was fivefold serially diluted (from 1000 to 8 μM) in reaction buffer and 50 μl aliquots added per well. After 5 min incubation at room temperature, 50 μl 1X target substrate DNA (TS DNA) solution was directly added to the 50 μl test sample within the wells. Reactions were mixed by tapping the plate gently 3–5 times and incubating for 30 min at 37 °C, washed 5 times with 300 μl wash solution and incubated with 100 μl HRP antibody for 30 min at 37 °C. After washing the plate under the same conditions, 100 μl TMB peroxidase substrate solution was added per well and incubated for 10 min at room temperature. To terminate the reaction, 100 μl TMB stop solution was directly added to wells and absorbance read using a plate reader at 450 nm.

### Confirmation of inhibition of 3′-processing activity of NCINI

To confirm the 3′-processing inhibition activity NCINI, the strand transfer inhibition assay was modified. The plate coating process with DS DNA was conducted in a similar manner. However, prior to treatment with LTR DS DNA, aliquots of fivefold serially diluted INI (from 2000 to 3.2 μM) were incubated with 20 nM IN for 30 min. Reaction of integrase first with the drug before its reaction with DS DNA is a necessary step to validate the 3′-processing inhibitory activity of the compound. The integrase-inhibitor mixture was added to LTR DS DNA-conjugated 96-well plates. Subsequent steps were conducted in a similar manner as the strand transfer assay.

### Statistical analysis

All measures of variance are presented as standard error of mean (SEM). Data were analyzed via two-way analysis of variance (two-way ANOVA) with Tukey post-hoc test using Prism8 (GraphPad Software, San Diego, CA). Differences were considered significant at *p*-values < 0.05.

## Results

### Comparison of INSTI and NCINI efficacy in the TZM-bl system

**Fig.1:**
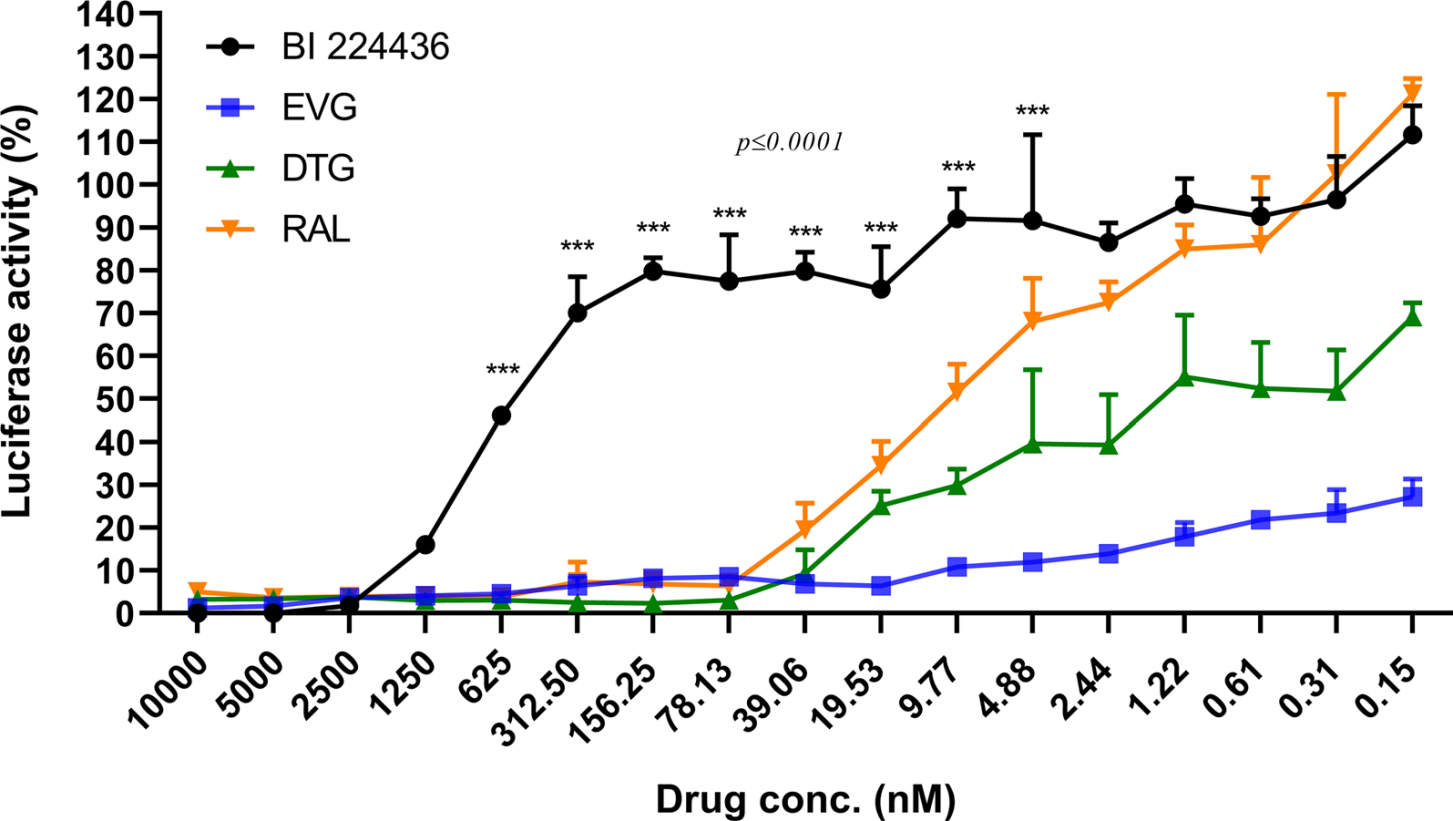
Determination of antiviral activities of integrase inhibitors in the TZM-bl cell line system. Integrase inhibitors were serially diluted and incubated with 1 × 10^4^ TZM-bl cells per well during 30 min, followed by infection with 500 TCID_50_ of HIV-1 AD8 strain. For determination of antiviral efficacy, luciferase activity was measured after 48 h of incubation. Cell culture media of non-infected and infected cells without HIV drug treatment were set as the negative and positive control, respectively. EVG, Elvitegravir; RAL, Raltegravir; DTG, Dolutegravir

TZM-bl cell lines expressing luciferase are commonly used for in vitro evaluation HIV associated [21]. We were commissioned to evaluate the effectiveness of NCINI candidates, but observed no activity using the TZM-bl system. BI 224436 was employed as the positive control in vitro to ascertain whether the TZM-bl system could be applied to assess the efficacy of NCINIs. Efficacy of candidates against HIV-1 AD8 was measured in TZM-bl cells (Fig. 1). EC_50_ values of 10.38 and 1.6 nM were obtained for RAL and DTG, respectively. EVG showed the greatest anti-HIV efficacy with an EC_50_ value < 0.15 nM. On the other hand, an EC_50_ value of 566.4 nM was obtained for BI 224436, suggestive of extremely low efficacy. And BI224436 data shows statistically significant differences with all INSTIs from 625 to 4.88 nM. In contrast to our data, BI 224436 is reported to have EC_50_ values in the range of 11–27 nM against HxB2, NL4.3 and a recombinant NL4.3 strain [19]. The extremely low antiviral activity of BI224436 evaluated using the TZM-bl system suggests that this assay may not be suitable for assessment of NCINI efficacy.

### Differential INSTI and NCINI activities using the TZM-bl and p24 ELISA assay systems

**Fig.2:**
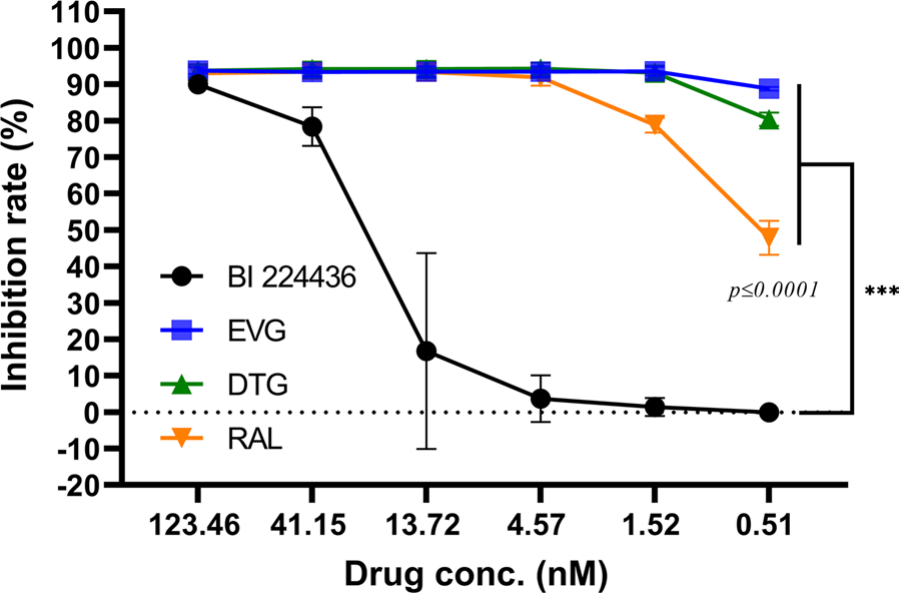
Comparison of antiviral activities of HIV drugs determined with p24 ELISA. MT2 cells infected with 500 TCID_50_ of HIV-1 AD8 strain were treated with a range of concentrations of each drug. At 5 days post-infection, cell culture media were harvested for p24 ELISA. Media of non-infected and infected cells without HIV drug treatment were set as the negative or positive control, respectively. EVG, Elvitegravir; RAL, Raltegravir; DTG, Dolutegravir

The capsid protein, p24, is the most abundant protein of HIV[22, 23] and commonly used in early detection and diagnosis of AIDS/HIV [23–26]. Given the low anti-HIV efficacy of BI22436 in our TZM-bl system, we performed a p24 ELISA assay to validate its antiviral activity and compared the results (Fig. 2). Because we considered p24 ELISA as the quickest and most convenient method to compare results. Notably, EC_50_ values of BI 224436, RAL, EVG, and DTG determined with p24 ELISA were 28.72, 0.58, > 0.51, and > 0.51 nM, respectively, distinct from data obtained with the TZM-b1 system.

### Comparison of cytotoxicity and SI value of INIs in the TZM-bl and MT2 cell

Evaluating the toxicity of drugs is one of the important factors in the development of new drugs. The selectivity index (SI) is defined as CC50/EC50 and it is a parameter of the efficacy and toxicity of the drug immediately. The ideal drug is to be toxic at high concentrations and effective at low concentrations. Thus, the higher the SI value theoretically, the safer and more effective the drug becomes [27, 28]. The cytotoxicity evaluation test was performed to calculate the SI value of each drug in both TZM-b1 (Table 1) and MT2 cell lines (Table 2) at 2 or 5 days after incubation, respectively. This incubation time is the same condition applied when efficacy test of a drug. BI 224436 displayed low cytotoxicity with high CC_50_ values > 50,000 nM in both MT2 and TZM-bl cell lines. In the TZM-b1 system, BI 224436 showed high EC_50_ and low SI values, it looks like unsuitable for further development (Table 1). On the other hand, in MT2 cells, BI 224436 showed a high SI value similar to EVG or DTG, indicating that the newly developed system is more appropriate for evaluating antiviral efficacy of NCINIs (Table 2).

**Table 1:** Cytotoxicity and effective concentrations of integrase inhibitors in the TZM-bl cell system

	CC50 (nM)	EC50 (nM)	SI value
BI 224436	> 50,000.00	566.40	> 88.28
EVG	26,041.67	< 0.15	> 173,611.13
RAL	> 50,000.00	10.38	> 4816.96
DTG	> 50,000.00	1.60	> 31,250.00

CC50, Half maximal cell cytotoxicity concentration; EC50, Half maximal effective concentration, SI value, Selectivity index, CC50/EC50

**Table 2:** Cytotoxicity and effective concentrations of integrase inhibitors in the MT2 cell system

	CC50 (nM)	EC50 (nM)	SI value
BI 224436	> 50,000.00	28.72	> 1740.95
EVG	1263.50	< 0.51	> 2477.46
RAL	6423.61	0.58	11,075.19
DTG	1740.93	< 0.51	> 3413.60

CC50, Half maximal cell cytotoxicity concentration; EC50, Half maximal effective concentration, SI value, Selectivity index, CC50/EC50

### Strand transfer inhibition test

**Fig.3:**
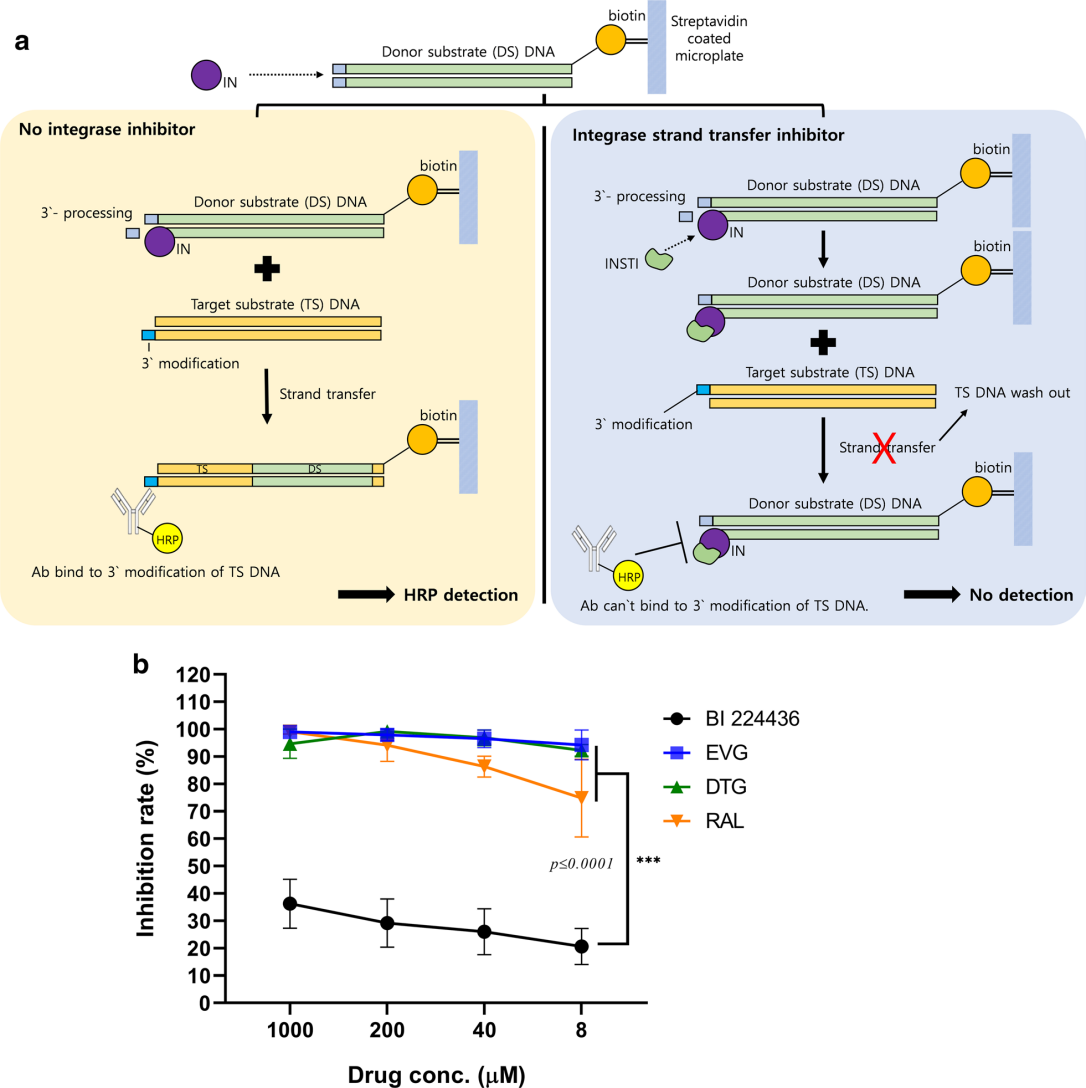
Strand transfer inhibition efficiency of catalytic and non-catalytic integrase inhibitors. **a** Schematic diagram of the strand transfer assay process. The yellow and blue boxes represent the response under conditions of no IN inhibitor or INSTI, respectively. **b** Integrase inhibitor compounds at a range of concentrations generated from fivefold serial dilutions were used to treat 3′-end cleaved LTR DS DNA by integrase. After incubation, TS DNA was added to each well for the strand transfer reaction. The reaction products were detected colorimetrically using a HRP-labeled antibody against modified target DNA. "No integrase" and "Integrase only" wells were set as the negative and positive control, respectively

To assess the differences in activities between NCINIs and INSTIs according to mechanism, an IN inhibition assay evaluating the effectiveness of strand transfer inhibition was performed according to original process (Fig. 3a). EVG, RAL, and DTG exerted > 50% inhibitory activity at 8 μM, with EVG exerting the greatest effect (up to 94% inhibition). In contrast, BI 224436 showed low inhibitory activity (36% at 1000 μM and 20% at 8 μM; Fig. 3b). Our data are consistent with previous reports that BI 224436 has an EC_50_ value > 50 μM and does not inhibit strand transfer [19, 20].

### Evaluation of differences in INSTI and NCINI activities according to inhibition mechanism

**Fig.4.**
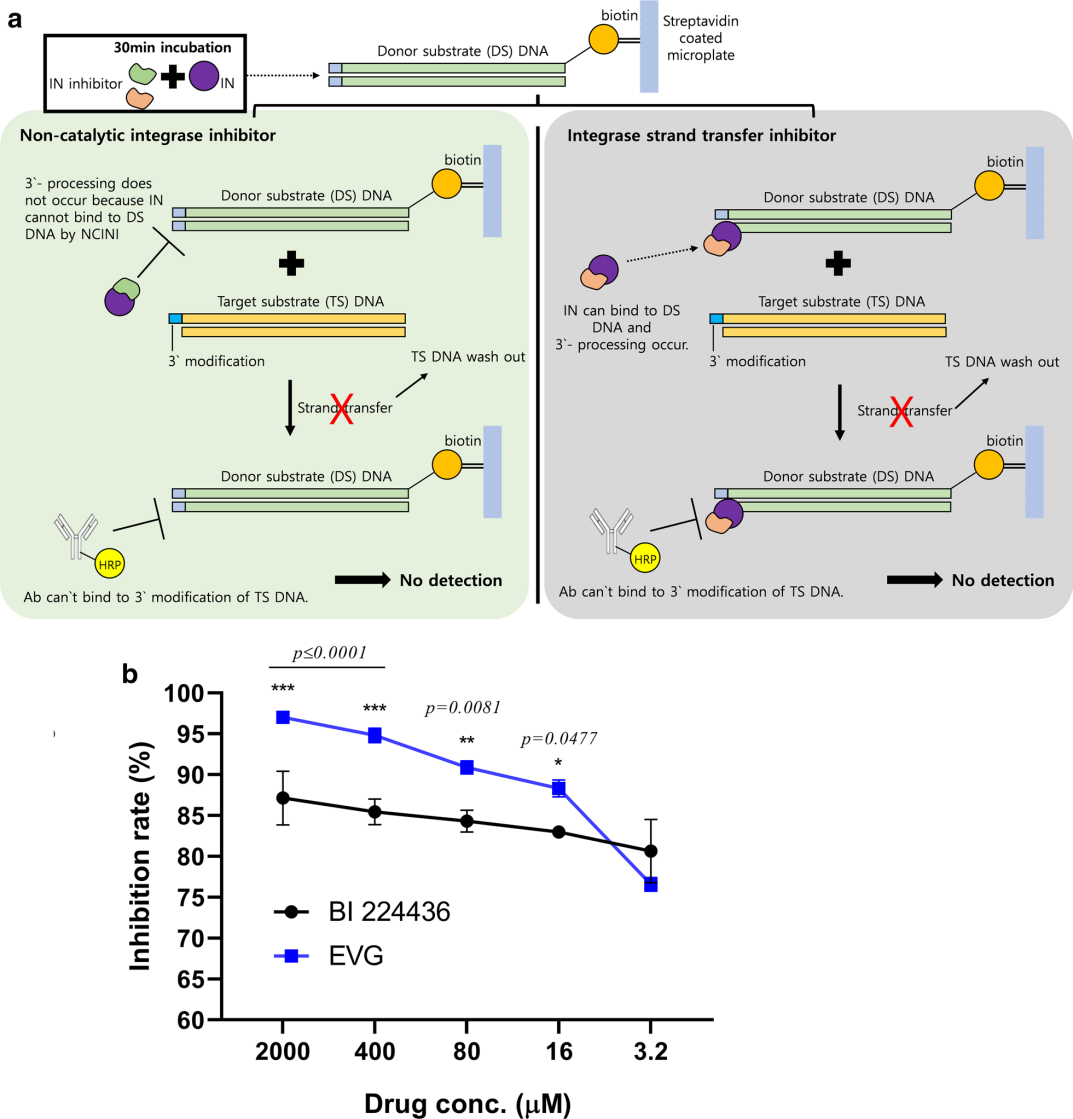
3′-Processing inhibition efficiency of catalytic and non-catalytic integrase inhibitors. **a** Schematic diagram of the 3′-processing inhibition assay. The 3′-processing inhibition assay was performed by modifying the protocol of the strand transfer assay. The green and gray boxes represent the response under conditions of NCINI or INSTI, respectively. **b** For determination of 3′-processing inhibition efficiency, the strand transfer assay protocol was modified. Before addition of LTR dsDNA, fivefold serially diluted integrase inhibitors were incubated with 20 nM integrase for 30 min. The integrase-inhibitor mixtures were treated with LTR dsDNA-conjugated 96-well plates. After incubation, target DNA was added to each well for the strand transfer reaction and the products detected colorimetrically using a HRP-labeled antibody against 3′-modified target DNA. "No integrase" and "integrase-only" wells were set as the negative and positive control, respectively

The experimental assay used in this study measured integrase inhibitor activity as follows: first, donor DNA was reacted with integrase that caused 3′-processing, followed by addition of inhibitor. Next, strand transfer was induced by adding target DNA. An HRP-conjugated antibody recognizing the 3′ modified target DNA was added to the mixture, and absorbance measured to determine the strand transfer inhibition rate. To assess 3′-processing inhibition by BI 224436 using this kit, the protocol was modified to allow initial reaction of the inhibitor with IN for 30 min, and the inhibitor-integrase mixture subsequently added to DS DNA. The subsequent steps were the same as the strand transfer assay protocol (Fig. 4a).

EVG showing the highest anti-HIV activity in the previous experiment was used as a control. Using the modified experimental protocol, BI 224436 showed significant IN inhibition activity at both high and low concentrations of 2000 and 3.2 μM (87.14% and 80.65%, respectively).

Additionally, high inhibitory activity of EVG (76.58–97.01%) was detected. Interestingly, BI 224436 was twice effective as an inhibitory agent in the modified system compared with original system. And at 3.2 μM, BI 224436 showed higher efficiency than EVG (Fig. 4b).

## Discussion

Following the earliest isolation and identification of HIV, extensive research has focused on effective treatments for the disease. The rapid development of drugs that inhibit HIV through various mechanisms, such as suppression of integration, reverse transcription and virus entry/fusion, has led to a steady decline in HIV-associated deaths worldwide. Evaluation of the efficacy of various types of drug candidates requires accurate methods based on their specific characteristics and mechanisms of action. Inappropriate analysis of candidates may lead to false or no results, and consequently, considerable losses to the drug development industry. We were commissioned to evaluate the efficacy of the novel NCINI candidates. Initially, efficacy assessment was conducted with the TZM-bl system, which we concluded was unsuitable for assessment of NCINI compounds in view of the low efficacy of BI 224436. Since we obtained different results compared to the previously known efficacy of BI224436 in our study using the TZM-bl system, we needed a clear and easy way to confirm this. In previous experiments, BI 224436 efficacy was evaluated using human peripheral blood mononuclear cells (hPBMCs), C8166 [19, 20], and T lymphocyte cell lines. Because hPBMCs are obtained by separating from normal human blood, there are more labor or procedural restrictions such as approval of institutional review board (IRB) than cell line in obtaining it. Furthermore, because p24 ELISA is a commonly used method in HIV diagnosis, the method met the condition we needed. For these reasons, the lymphocyte cell line was employed to facilitate evaluation of the process for p24 ELISA in our experiment. We performed a p24 ELISA method by infecting the suspension cell with the virus for longer periods of time, which yielded distinct results from the TZM-bl system. p24 ELISA result obtained using MT2 cells showed nearly 20-fold higher inhibition activity than those with the TZM-bl system, consistent with earlier findings on BI 224436 [19, 20]. INSTI compounds used as controls showed effective anti-HIV activity in both TZM-b1 and p24 ELISA assays while accurate anti-HIV activity of BI 224436 was only determined via p24 ELISA. The key difference between the two systems is the possibility of long-term incubation after virus infection or drug treatment, which is attributable to the different cell lines used [29].

Finally, the 3′-processing inhibition activity of BI224436 was also confirmed through the modified process.

NCINI is classified as an allosteric integrase inhibitor (ALLINI) [13]. ALLINI directly inhibits not only HIV integration but also binding of viral RNA with integrase in virions. The compound causes damage in the morphogenesis process, creating a non-infectious virion. Reportedly, NCINI exerts its inhibitory activity under conditions of more than two cycles of virus replication that takes more than four days [29]. Thus, we hypothesized the incubation time of cells in efficacy analysis of NCINI may be an important factor for accurate activity measurement. TZM-bl is an adherent cell line and due to spatial constraints, long-term monitoring is not possible owing to cell overgrowth problems following incubation periods of more than three days. However, the suspension cell such as MT2 can be cultured for longer periods (more than four days) compared to adherent cell lines due to less restriction of space. Moreover, since HIV replication and whole life cycle can be fully achieved in the MT2 cell that differentiates with TZM-bl cell, the antiviral activity of allosteric integrase inhibitors may be maximized. Therefore, when assessing the efficacy of NCINI and other ALLINI candidates in cells, the precise effects may be measurable only under incubation conditions of more than four days. It would also be very important to use a cell line that HIV can multiply. Some ALLINIs bind directly to CCD of IN to exert their effects but others are reported to inhibit protein–protein interactions by combining it into a distinct site [13, 19, 29]. BI 224436, the first identified NCINI, prevents integration by blocking 3′-processing as well as structural deformation of CCD. Therefore, for evaluation of the effectiveness of new ALLINI candidates, co-execution of cell and mechanism-based activity assays, such as the 3′-processing inhibition assay, should be considered.

## Conclusion

Herein, we have demonstrated differences in NCINI efficacy based the evaluation method and developed a novel protocol to facilitate accurate evaluation of antiviral activity. For development of NCINI as a new class of drug, accurate drug efficacy assessment is urgently required to allow drug regulators to make informed decisions.[30–32] Our study presents a novel assay method for NCINI activity through simple modification of the existing protocol and may serve as a guideline for clinical efficacy assessment of NCINI compounds in the future.

## Data Availability

All data generated or analyzed during this study are included in this published article.

## Abbreviations

AIDS: Acquired immune deficiency syndrome
ALLINI: Allosteric integrase inhibitor
CCD: Catalytic core domain
DTG: Dolutegravir
EVG: Elvitegravir
FDA: US Food and Drug Administration
HAART: Highly active anti-retroviral therapy
hPBMC: Human peripheral blood mononuclear cell
IN: Integrase
INI: Integrase inhibitor
INSTI: Integrase strand transfer inhibitor
LEDGF/p75: Lens epithelial-derived growth factor/p75
LTR: Long-terminal repeat
NCINI: Non-catalytic integrase inhibitor
NNRTI: Non-nucleoside reverse transcriptase inhibitor
NRTI: Reverse transcription inhibitor
PI: Protease inhibitor
PIC: Pre-integration complex
RAL: Raltegravir
RTI: Reverse transcription inhibitor
